# A dynamic body-selective area localizer for use in fMRI

**DOI:** 10.1016/j.mex.2020.100801

**Published:** 2020-01-23

**Authors:** Paddy Ross, Beatrice de Gelder, Frances Crabbe, Marie-Hélène Grosbras

**Affiliations:** a Department of Psychology, Durham University, Durham, UK; b Institute of Neuroscience and Psychology, University of Glasgow, Glasgow, UK; c Department of Cognitive Neuroscience, Maastricht University, Maastricht, the Netherlands; d Laboratoire De Neurosciences Cognitives, Aix Marseille Université, Marseille, France

**Keywords:** Dynamic body-selective area localizer, fMRI, Body perception, Emotion, Dynamic bodies, Body-selective areas

## Abstract

Functional localizers allow the definition of regions of interest in the human brain that cannot be delineated by anatomical markers alone. To date, when localizing the body-selective areas of the visual cortex using fMRI, researchers have used static images of bodies and objects. However, there are other relevant brain areas involved in the processing of moving bodies and action interpretation that are missed by these techniques. Typically, these biological motion areas are localized separately using whole and scrambled point-light display stimuli. Currently, one can only localize *either* the static body-selective areas *or* the biological motion areas, but not both together. Here, for the first time, using motion-controlled dynamic body and object stimuli, we describe a method for localizing the full dynamic body-selective network of the human brain in one experimental run.

• The method uses dynamic body and object stimuli.
• Low-level local motion information is added as a covariate into the fMRI analysis.
• This localizes the full dynamic body-selective network of the human brain.

## Graphical abstract

**Figure:**
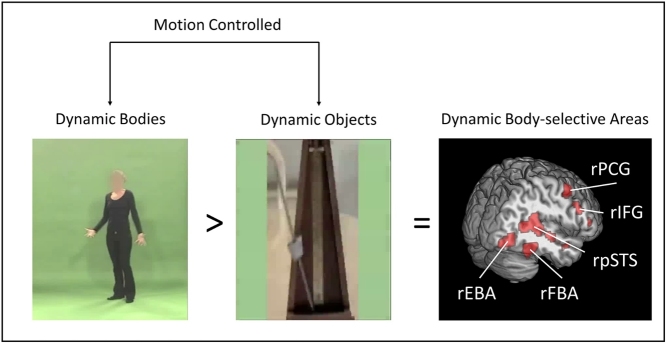

**Specification Table**

**Table:** 

Subject Area:	Neuroscience
More specific subject area:	Human body perception
Method name:	Dynamic body-selective area localizer
Name and reference of original method:	Body-Selective Area localizer using static images of bodies and objects
Resource availability:	Stimuli and code available from https://lnc.univ-amu.fr/fr/profile/grosbras-marie-helene.

## Method details

### Background

The use of functional localizers in functional magnetic resonance imaging (fMRI) allows researchers to define regions of interest (ROIs) which cannot be delineated by anatomical markers alone. The anatomical landmarks and their respective functional activations are highly variable across individuals [1,2], thus isolating them in individual subjects allows for direct group-level comparison. Using this approach, localizers have been used to characterize a large number of brain regions, including ROIs which show selective response to vocalizations [3], higher-level language processing [4] and the written word [5], static faces [6], and static images of bodies [7].

However, while the vocal, language and face related localizers use stimuli which one may encounter in everyday life, one is unlikely to ever observe static bodies. It is known that viewing dynamic bodies elicits response in the posterior superior temporal sulcus (pSTS) [[8], [9], [10], [11]] and inferior frontal gyrus (IFG) [[12], [13], [14], [15]]. In contrast to the extra-striate (EBA) and fusiform (FBA) body areas (which show increased activation towards bodies compared with objects regardless of whether or not the stimuli are moving), activity in the pSTS is thought to be related only to bodies in motion and is crucial to the detection of socially relevant information concerning others’ actions [16]. These areas are therefore key ROIs in the body processing networks, and currently are not identifiable by standard static body localizers.

To date, these biological motion regions have been localized separately from the body-selective areas by using point-light display (PLD) stimuli [17,18]. Biological motion by way of PLDs is presented and contrasted with scrambled motion to localize the pSTS, but this is very rarely linked with the body-selective ROIs (FBA, EBA etc.). As a result, currently, one can only localize *either* the static body-selective areas *or* the biological motion areas, but not both together (see Table 1 for current state of the literature). Here, therefore, by controlling for low-level local visual motion across stimuli and adding this measure as a covariate to our fMRI analysis we present a method of localizing the entire dynamic body-selective network in the visual cortex (body-selective and biological motions areas) using dynamic full-body stimuli contrasted against dynamic objects in one scan [19,20].

**Table 1:** Body-Selective and Body Motion areas localized by other select work in the literature. FBA = Fusiform Body Area; EBA = Extra-striate Body Area; STS = Superior Temporal Sulcus; IFG = Inferior Frontal Gyrus; PCG = Precentral Gyrus.

	Brain Areas Localized						
	Body-Selective Areas			Body Motion Areas			
Static Bodies	rEBA	rFBA	lEBA	rpSTS	lpSTS	rPCG	rIFG
Downing et al. [7] Static Body > Objects	✓		✓				
Grossman & Blake [17] Static Bodies > Objects	✓		✓				
Peelen & Downing [21] Stick Bodies > Scrambled Bodies	✓	✓					
Peelen et al. [22] Static Bodies > Objects	✓	✓	✓				
Peelen et al. [23] Static Bodies > Tools	✓	✓	✓				
Brandman & Yovel [24] Static Bodies > Inverted Bodies	✓	✓	✓				
Kret et al. [25] Static Bodies > Houses	✓	✓	✓				
Brandman & Yovel [26] Whole Bodies > Body Parts	✓	✓					

Dynamic PLDs							
Grossman et al. [27] Dynamic PLDs > Scrambled PLDs				✓			
Grossman & Blake [17] Dynamic PLDs > Scrambled PLDs				✓	✓		
Saygin et al. [28] Dynamic PLDs > Scrambled PLDs				✓		✓	✓
Peelen et al.[ 22] Dynamic PLDs > Scrambled PLDs				✓			
Jung et al. [29] Dynamic PLDs > Scrambled PLDs				✓	✓	✓	✓
Atkinson et al. [30] Dynamic PLDs > Scrambled PLDs				✓			

Dynamic Bodies (Current Method Described)							
Ross et al. [20] Dynamic Bodies > Dynamic Objects (Motion controlled)	✓	✓	✓	✓	✓	✓	✓

### Stimuli

Forty-five short video-clips were taken from a larger set created and validated by Kret et al. [25]. Each clip depicted one actor, dressed in black against a green background, moving in an angry, happy or neutral manner. Six actors were males and nine females, with each actor recorded three times for each of the three emotions. The videos were recorded using a digital video camera and were edited to two-second long clips (50 frames at 25 frames per second). The faces in the videos were masked with Gaussian filters so that only information from the body was perceived (for full details and validation of stimuli (see Kret et al. [25] de Gelder and Van den Stock [31]). In addition, to use as control stimuli, we selected videos depicting non-human moving objects (e.g. windscreen wipers, windmills, metronomes etc.) from the internet. We edited these clips using Adobe Premiere so that they matched the body stimuli in terms of size and resolution (960 × 540 pixels). A green border matching the color (RGB: 159, 202, 145) of the human video background was added and stimuli were presented in blocks of five clips (10 s). A sample of the stimuli used can be found in the supplementary material, and the full stimuli set and localizer code is available at https://lnc.univ-amu.fr/fr/profile/grosbras-marie-helene.

Furthermore, to control for potential low-level parameters effects on fMRI activity, we computed one measure of low-level local visual motion in the clips in order to enter it as a covariate in our fMRI regression analysis. In each clip, we first calculated frame-to-frame change in luminance in the background as a surrogate measure of noise level. Then for each pair of consecutive frames we counted the number of pixels where the change in intensity was higher than noise. We averaged these numbers yielding one value per clip, representing the motion in this clip. This measure showed a high correlation with measures of perceived motion in the clips as rated by a group of healthy adults (r = .571, n = 61, *p* < .001). For the localizer we computed the cumulative motion for the five clips in each block of the experimental design. Overall the blocks of non-human clips were not significantly different than the body movement clips (*t*(16) = 1.89, *p* = 0.076). Nevertheless, to control for any potential effect of motion these measures were added as a covariate in our fMRI analysis.

### Experimental design

The localizer was programmed with MATLAB using the Psychophysics Toolbox Extensions [32]. An experimental run consisted of 48 10-seconds long blocks: eighteen blocks of non-human stimuli (10 s; 5 clips), eighteen blocks of human stimuli (three blocks of each emotion) and twelve 10-seconds-long blocks of blank screen as a baseline, in a pseudo-randomized order based on an m-sequence avoiding correlation effects between blocks [33].

### Data acquisition

We acquired a series of 246 images of brain activity using a 3 T fMRI scanner (Tim Trio, Siemens, Erlangen, Germany) equipped with a 32-channels head coil, using standard EPI sequence for functional scans (TR/TE: 2600 ms/40 ms; slice thickness = 3 mm; in plane resolution = 3 × 3 mm). In addition, we performed a high-resolution T1-weighted structural scan (1 mm3 3D MPRAGE sequence) for anatomical localization.

### Pre-processing

We used SPM 8 (Welcome Department of Imaging Neuroscience; www.fil.ion.ucl.ac.uk/spm) to process and analyse the fMRI data. The functional data were corrected for motion by using a two-pass procedure. First we estimated the rigid-body transformation necessary to register each image to the first one of the time series and applied this transformation with a 4th Degree B-Spline interpolation. Then we averaged all these transformed images and repeated the procedure to register all images to the mean image. Movement correction was allowed up to 2 mm translation or 2 degrees rotation. The realigned functional data were co-registered with the individual 3D T1-weighted scan. First we identified AC-PC landmarks manually and estimated the affine transformation from the mean functional image to the structural image. Then this transformation was applied to the whole realigned time series.

The anatomical scans were then segmented for different tissue types and transformed into MNI-space using non-linear registration. The parameters from this transformation were subsequently applied to the co-registered functional data. Before performing the analyses, we smoothed the data using a Gaussian kernel (8 mm FWHM). High-pass temporal filtering was applied at a cut off of 128 s to remove slow signal drifts.

## Method validation

A sample of 26 adults recruited from the University of Glasgow took part in the localizer validation (age 18–27: M = 21.28 years; SD = 2.11, 15 female). Participants were installed comfortably in the scanner. Head motion was restricted by comfortable but tight padding. Stimuli were back-projected onto a screen positioned at the back of the scanner bore. Participants were able to view the screen thanks to a mirror attached to the head-coil. They were instructed to maintain their gaze in the center of the screen. Before the main experiment started they were reminded to pay careful attention to the stimuli, to look at the central fixation cross and to keep their head still.

### Whole brain analysis

A general linear model was created with one predictor for body and one for non-body conditions. We added our measure of luminance change (video clip motion) as a covariate, allowing us to control for mild differences in motion. The six head-motion parameters were also added as regressors of non-interest. The model was estimated for each participant and we computed the Body > Non-Human contrast of interest between individual parameter estimates. These contrast images were taken to a second-level random effect analysis of variance (ANOVA) to create group-averages. For the localizer, resulting statistical maps are presented using a threshold of *p* < 0.05 after Familywise error (FWE) correction at the voxel level and a cluster extent of a minimum of 10 voxels. Anatomical locations for the peak functional activations were determined with the help of the Harvard-Oxford cortical and sub-cortical structural atlases as implemented in FSLview [34]. In addition, we also examined results at the individual level. At *p* < .001 uncorrected threshold we observed activity in 80 % of participants in all regions but the right IFG (70 %). At a more stringent threshold of *p* < .05 FWE corrected we still observed activation in over 80 % of participants in the rFBA, rEBA, rpSTS, 70 % in the lEBA, 65 % in the PCG, 58 % in the lpSTS and 27 % of participants showed rIFG activation. At the threshold used in [22] (p<0.05 uncorrected) we observed activity in 100 % of participants in the rFBA, rEBA, rpSTS, rPCG, 96 % in the lpSTS, 92 % in the lEBA and 88 % in the rIFG.

**Fig. 1:**
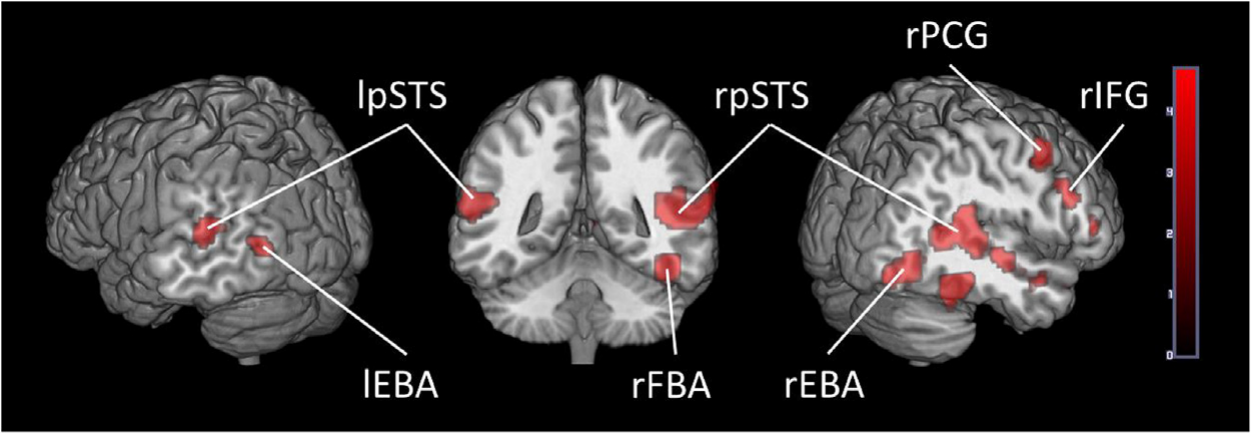
Brain activity when contrasting Dynamic Bodies > Dynamic Objects in Adults. (p < 0.05 FWE corrected, cluster extent threshold of 10 voxels). Color-bar indicates the threshold of the t-value.

Our localizer results are presented in Table 2, Table 3 and Fig. 1 below. We also present in Table 4 a comparison of the average coordinates in our subjects using the current localizer with the averages from the literature described in Table 1.

**Table 2:** Regions activated in whole-brain group-average random-effects analysis contrasting Bodies > Non-Bodies (p < 0.05 FWE corrected, cluster extent threshold of 10 voxels, maximum cluster sphere 10 mm radius). Coordinates are in MNI space. Numbers of participants showing significant activation and the standard deviation of the number of activated voxels in each ROI are also presented. FBA = Fusiform Body Area; EBA = Extra-striate Body Area; STS = Superior Temporal Sulcus; IFG = Inferior Frontal Gyrus; PCG = Precentral Gyrus.

Group Level						
Region	x	y	z	Peak-*t*	No. of Voxels p < .05 FWE corr.	No of Voxels p < .001 uncorr.
rFBA	45	−46	−17	12.6	91	141
rEBA	45	−79	−11	11.8	74	104
rpSTS	54	−46	7	10.41	124	162
rPCG	48	5	46	9.41	46	148
lEBA	−48	−73	7	7.9	25	120
lpSTS	−63	−49	19	7.6	42	118
rIFG	42	20	22	7.8	32	136

**Table 3:** ROIs at an individual level random-effects analysis contrasting Bodies > Non-Bodies (p < 0.05 FWE corrected, p < .001 uncorrected and p < .05 uncorrected). Mean coordinates are in MNI space. Numbers of participants showing significant activation and average number of activated voxels in each ROI are presented at all thresholds. FBA = Fusiform Body Area; EBA = Extra-striate Body Area; STS = Superior Temporal Sulcus; IFG = Inferior Frontal Gyrus; PCG = Precentral Gyrus.

Individual Level				P < .05 FWE Corr.		P < .001 Uncorr.		P < .05 Uncorr	
Region	x M (SD)	y M (SD)	z M (SD)	No. showing activation /26	No. of Voxels	No. showing activation /26	No. of Voxels	No. showing activation /26	No. of Voxels
rFBA	42(2.0)	−44(2.6)	−19(2.6)	21	16	25	28	26	41
rEBA	49(3.5)	−71(3.3)	−3(3.8)	24	41	26	53	26	66
rpSTS	56(3.6)	−56(5)	11(2.8)	23	35	24	49	26	63
rPCG	49(3.2)	5(3.0)	46(4.9)	17	23	22	42	26	55
lEBA	−48(3.4)	−75(3.4)	−2(3.5)	18	25	21	33	24	39
lpSTS	−46(3.5)	−56(5.2)	13(2.8)	15	14	24	19	25	41
rIFG	46(4.0)	18(3.1)	23(3.7)	7	18	18	26	23	54

**Table 4:** Average and SD of coordinates from individual subjects in the current localizer compared with the average and SD of coordinates from localizers in the literature described in Table 1. Coordinates are in MNI space.

Current Method Described				Average From Literature		
Region	x	y	z	x	y	z
rFBA	42(2)	−44(3)	−19(3)	42(1)	−44(2)	−24(3)
rEBA	49(4)	−71(3)	−3(4)	47(4)	−69(2)	0(4)
rpSTS	56(4)	−56(5)	11(3)	54(4)	−52(7)	12(4)
rPCG	49(3)	5(3)	46(5)	43(6)	9(2)	42(11)
lEBA	−48(3)	−75(3)	−2(4)	−47(5)	−74(2)	4(5)
lpSTS	−46(4)	−56(5)	13(3)	−49(6)	−57(4)	16(8)
rIFG	46(4)	18(3)	23(4)	44(8)	14(3)	15(7)

## Conclusions

Currently, a localizer for the dynamic body-selective areas is missing from the literature. Here, for the first time, we present a localizer using motion controlled dynamic bodies and objects that can be used to localize both the body-selective and body-motion areas at the same time. Furthermore, by adding the low-level motion information from the stimuli into the fMRI model as a covariate, we also control for any activity that could be caused by unwanted differences in motion across stimuli. We believe that this approach will be of great interest to the wider community. It will provide a time-efficiency advantage for those who are studying specifically body perception in different conditions; furthermore it would provide a standard tool to compare different populations, as we did in our developmental study [20], but potentially also in clinical populations. This method will complement advantageously the paradigms for testing social perception, which are either too often centered on higher processes or, when concerned with primary perceptual processes, focus solely on face perception.

## Declaration of Competing Interest

The author declares that the research was conducted in the absence of any commercial or financial relationships that could be construed as a potential conflict of interest.
